# Impacts of heat exposure in utero on long-term health and social outcomes: a systematic review

**DOI:** 10.1186/s12884-024-06512-0

**Published:** 2024-05-04

**Authors:** Nicholas Brink, Darshnika P. Lakhoo, Ijeoma Solarin, Gloria Maimela, Peter von Dadelszen, Shane Norris, Matthew F. Chersich, Climate and Heat-Health Study Group, Admire Chikandiwa, Britt Nakstad, Caradee Y. Wright, Lois Harden, Nathalie Roos, Stanley M. F. Luchters, Cherie Part, Ashtyn Areal, Marjan Mosalam Haghighi, Albert Manyuchi, Melanie Boeckmann, Minh Duc Pham, Robyn Hetem, Dilara Durusu

**Affiliations:** 1 Climate and Health Directorate, Wits RHI, University of the Witwatersrand, Johannesburg, South Africa; 2 King’s College, London, United Kingdom; 3 MRC Developmental Pathways for Health Research Unit, University of the Witwatersrand, Johannesburg, South Africa

**Keywords:** Climate change, Heat exposure, Pregnancy, Long-term effects, Socioeconomic impact, Maternal health, Child health, Epigenetics, Metabolic disease

## Abstract

**Background:**

Climate change, particularly global warming, is amongst the greatest threats to human health. While short-term effects of heat exposure in pregnancy, such as preterm birth, are well documented, long-term effects have received less attention. This review aims to systematically assess evidence on the long-term impacts on the foetus of heat exposure in utero.

**Methods:**

A search was conducted in August 2019 and updated in April 2023 in MEDLINE(PubMed). We included studies on the relationship of environmental heat exposure during pregnancy and any long-term outcomes. Risk of bias was assessed using tools developed by the Joanna-Briggs Institute, and the evidence was appraised using the GRADE approach. Synthesis without Meta-Analysis (SWiM) guidelines were used.

**Results:**

Eighteen thousand six hundred twenty one records were screened, with 29 studies included across six outcome groups. Studies were mostly conducted in high-income countries (*n* = 16/25), in cooler climates. All studies were observational, with 17 cohort, 5 case-control and 8 cross-sectional studies. The timeline of the data is from 1913 to 2019, and individuals ranged in age from neonates to adults, and the elderly. Increasing heat exposure during pregnancy was associated with decreased earnings and lower educational attainment (*n* = 4/6), as well as worsened cardiovascular (*n* = 3/6), respiratory (*n* = 3/3), psychiatric (*n* = 7/12) and anthropometric (*n* = 2/2) outcomes, possibly culminating in increased overall mortality (*n* = 2/3). The effect on female infants was greater than on males in 8 of 9 studies differentiating by sex. The quality of evidence was *low* in respiratory and longevity outcome groups to *very low* in all others.

**Conclusions:**

Increasing heat exposure was associated with a multitude of detrimental outcomes across diverse body systems. The biological pathways involved are yet to be elucidated, but could include epigenetic and developmental perturbations, through interactions with the placenta and inflammation. This highlights the need for further research into the long-term effects of heat exposure, biological pathways, and possible adaptation strategies in studies, particularly in neglected regions. Heat exposure in-utero has the potential to compound existing health and social inequalities. Poor study design of the included studies constrains the conclusions of this review, with heterogenous exposure measures and outcomes rendering comparisons across contexts/studies difficult.

**Trial Registration:**

PROSPERO CRD 42019140136.

**Supplementary Information:**

The online version contains supplementary material available at 10.1186/s12884-024-06512-0.

## Introduction

Climate change is one of the most significant threats to human health [1], characterized by an increase in global temperatures amongst other environmental changes. Global temperatures have increased by approximately 1·2 °C, and are projected to increase beyond a critical threshold of 1·5 °C in the next 5–10 years [2]. Increasingly, heat exposure is being linked with a multitude of short- and long-term health effects in vulnerable populations, including children [3], the elderly, and pregnant women [4]. The effect on pregnant women extends to the health of the foetus, with significant detrimental effects associated with heat exposure including preterm birth, stillbirth, and decreased birth weight [5]. Impacts of heat exposure are increasingly important in populations in resource-constrained settings, where heat adaptation measures such as active (air-conditioning) and passive cooling (water, green and blue spaces) are limited, and often inaccessible [6]. These populations are often found in some of the hottest climates and in areas whose contribution to global warming is negligible, thus compounding inequities [7]. In addition, research in this field is biased towards Europe, North America and Asia and is profoundly underrepresented in Africa and South America [8]. Understanding the scope and distribution of research conducted is key to guiding future research, including biological studies to explore possible mechanisms, and interventional studies to alleviate any observed negative effects. Multiple previous systematic reviews have explored the short-term impacts of heat on the foetus [3, 5, 9] but only one has explored the long-term impacts of heat exposure on mental health [10]. The in-utero environment has long been considered important in the long-term health and wellbeing of individuals [11, 12], although it has been challenging to delineate specific causal pathways. This study aims to systematically review the literature on the long-term effects of heat exposure in-utero on the foetus, and explore possible casual pathways.

## Materials and methods

This review forms part of a larger systematic mapping survey of the effect of heat exposure, and adaptation interventions on health (PROSPERO CRD 42019140136) [13]. The initial literature search was conducted in September 2018, where the authors searched MEDLINE (PubMed), Science Citation Index Expanded, Social Sciences Citation Index, and Arts and Humanities Citation Index using a validated search strategy (Supplementary Text 1). This search was updated in April 2023 through a MEDLINE search, as all previous articles were located in this database. Screening of titles and abstracts was done independently in duplicate, with any differences reconciled by MFC, with subsequent updates conducted by NB and DL. The authors only included studies on humans, published in Chinese, English, German, or Italian. Studies on heat exposure from artificial and endogenous sources were excluded, and only exogenous, weather-related heat exposure during pregnancy was included. All study designs were eligible except modelling studies and systematic reviews. No date restrictions were applied. EPPI-Reviewer software [14] provided a platform for screening, reviewing of full text articles, and for data extraction. No additional information was requested or provided by the authors. Long-term effects were defined as any outcomes that were not apparent at birth.

Articles meeting the eligibility criteria were extracted in duplicate after the initial search and then by a single reviewer in the subsequent update (NB/DL). Data were extracted to include characteristics outlined in Supplementary file 1.

This systematic review was conducted according to the Systematic Review without Meta-Analysis (SWiM) guidelines, broadly based on PRISMA [15], as the outcomes, statistical techniques, and heat exposure measurements were heterogenous, rendering a meta-analysis untenable. Outcomes were grouped clinically, reviewed for the magnitude and direction of effect, and their statistical significance, and included negative or null findings when reported on. A text-based summary of these findings was made. ‘Vote-counting’ was utilized to summarise direction of effect findings. Analysis was conducted on the geographical areas, climate zones [16], mean annual temperature and socioeconomic classification of the country where the studies were conducted. Furthermore, an attempt was made to identify at-risk population sub-groups.

The principal investigator assessed each study for a risk of bias using the tools developed by the Joanna-Briggs Institute (JBI) [17] (Supplementary file 1). Each study was classified as high or low risk of bias. Studies that did not score ‘yes’ on two or more applicable parameters were classified as high risk of bias [5]. Due to the limited research in this field, no studies were excluded based on risk of bias. The certainty of the evidence was assessed using the GRADE approach, with the body of evidence assessed on a scale of certainty: *very low, low, moderate* and *high *[18]. Due to the heterogeneity of outcomes, and the reporting thereof, assessment of publication bias was not possible.

The funder of the study had no role in study design, data collection, analysis, interpretation, or writing of the report.

## Results

**Fig. 1:**
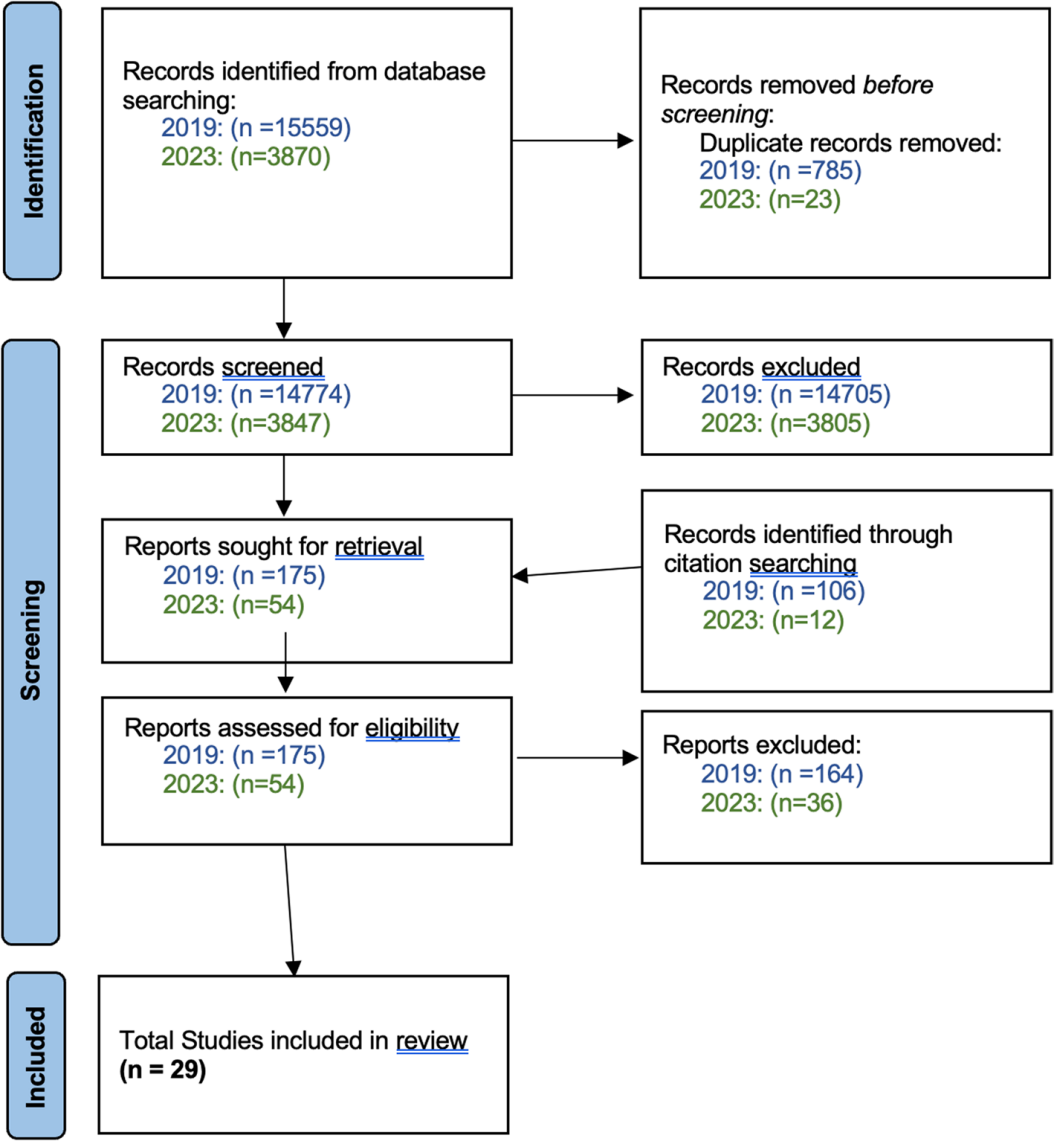
PRISMA flow diagram

**Fig. 2:**
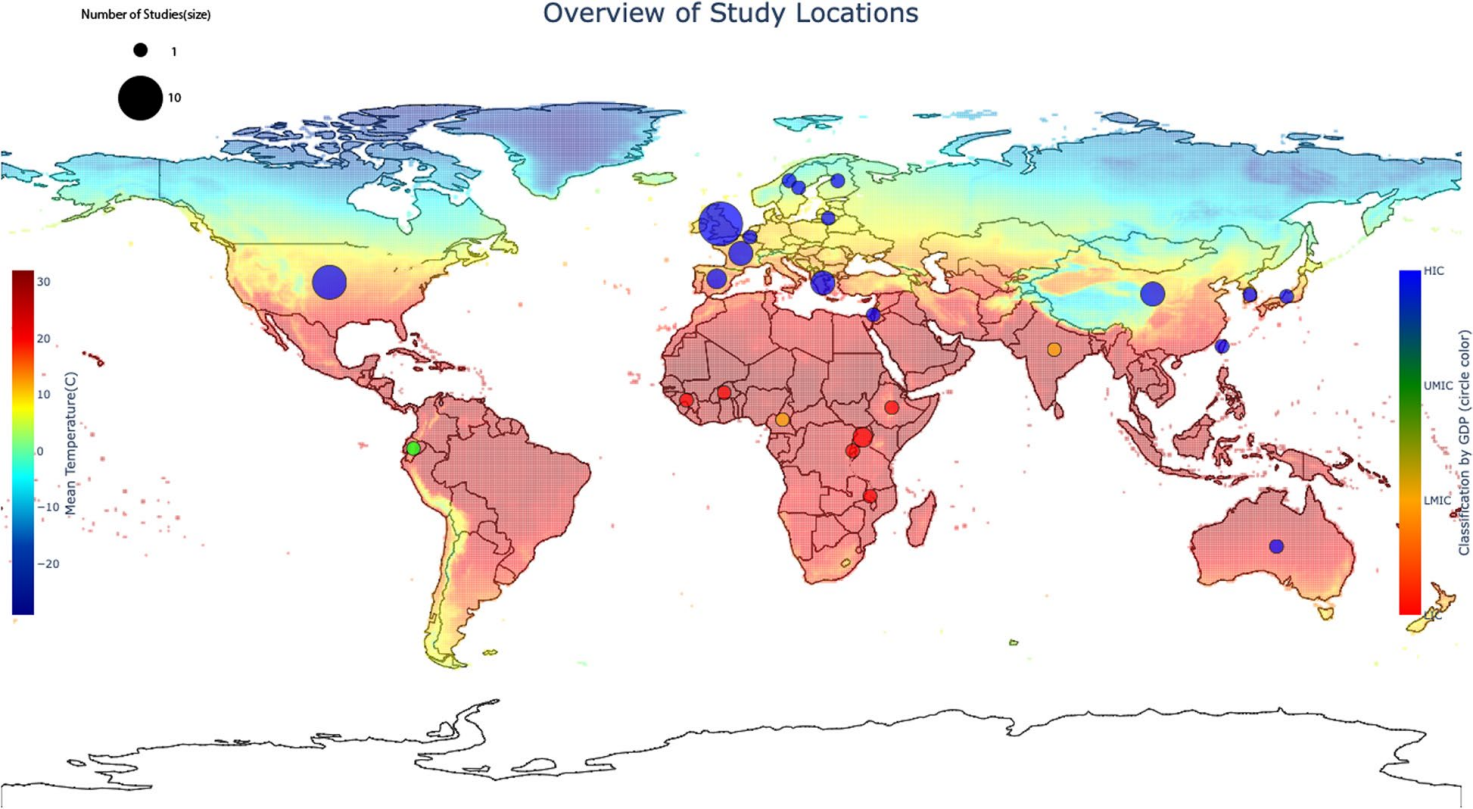
Map showing countries where studies were conducted relative to mean annual temperature [21]

**Fig. 3:**
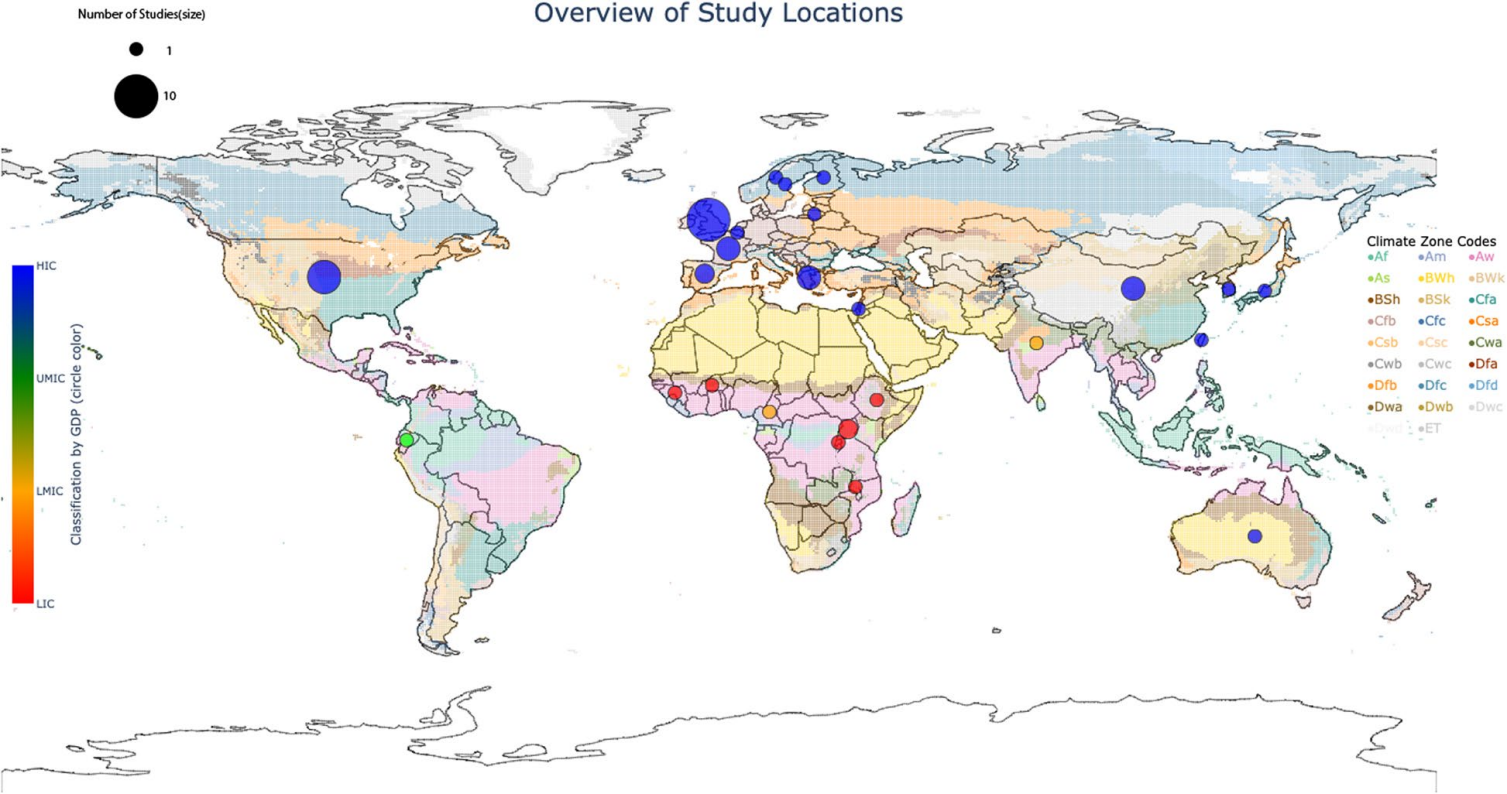
Map showing countries where studies were conducted relative to climate zones [16]

The updated search identified 18 621 non-duplicate records, and after screening 229 full-text articles were reviewed for inclusion, with a total of 29 studies included in the final analysis (Fig. 1: flow chart). The included studies were conducted in 25 countries across six continents, including six Low-Income Countries (LIC), two Lower-Middle Income Countries (LMIC), one Upper-Middle Income Country (UMIC) and 16 High Income Countries (HIC) [19]. They included 25 Köppen-Geiger climate zones [16], and mean annual temperatures ranging from 2.1 °C in Norway to 30.0 °C in Burkina Faso [20] (Figs. 2 and 3). All studies were observational, with 17 cohort, five case-control and eight cross-sectional studies. The timeline of the data is from 1913 to 2019, and individuals included ranged in age from neonates to adults, and the elderly. The studies were grouped by outcomes as follows: behavioural, educational and socioeconomic (*n* = 6), cardiovascular disease (*n* = 6), respiratory disease (*n* = 3), growth and anthropometry (*n* = 2), mental health (*n* = 12) and longevity and mortality (*n* = 3). The measures of heat exposure were variable, with minimum, mean, maximum, and apparent temperature being utilized, as well as temperature variability, heat wave days and discreet shocks (number of times exposure exceeded a specific threshold). The majority of studies measured heat using mean temperature (*n* = 27/29). In addition, the statistical comparison was diverse, with some studies making a continuous linear comparison by degree Celsius, while others compared heat exposure by quartiles, amongst other categorical comparisons. Furthermore, heat exposure by any definition was not reported over the same timeframes, with some studies including variable periods before birth, during pregnancy and at birth in their analysis. Levels of temporal resolution of heat exposure were also diverse, ranging from monthly effects to effects observed over the entire gestational period, or year of birth. In addition, differing use of heat adaptation mechanisms was not uniformly described and adjusted for. Various confounders were adjusted for, and although not uniform, these were generally inadequate. The effect on female infants was greater than on male infants in eight of nine studies differentiating by sex, with increased effects on marginalised groups (African-Americans) in one further study. Overall, the quality of the evidence, as assessed by the GRADE approach, was *low* in respiratory and longevity outcome groups to *very low* in all other groups, primarily as a result of their observational nature and high risk of bias, due to insufficient consideration of confounders, and inadequate measures of heat exposure.

**Fig. 4:**
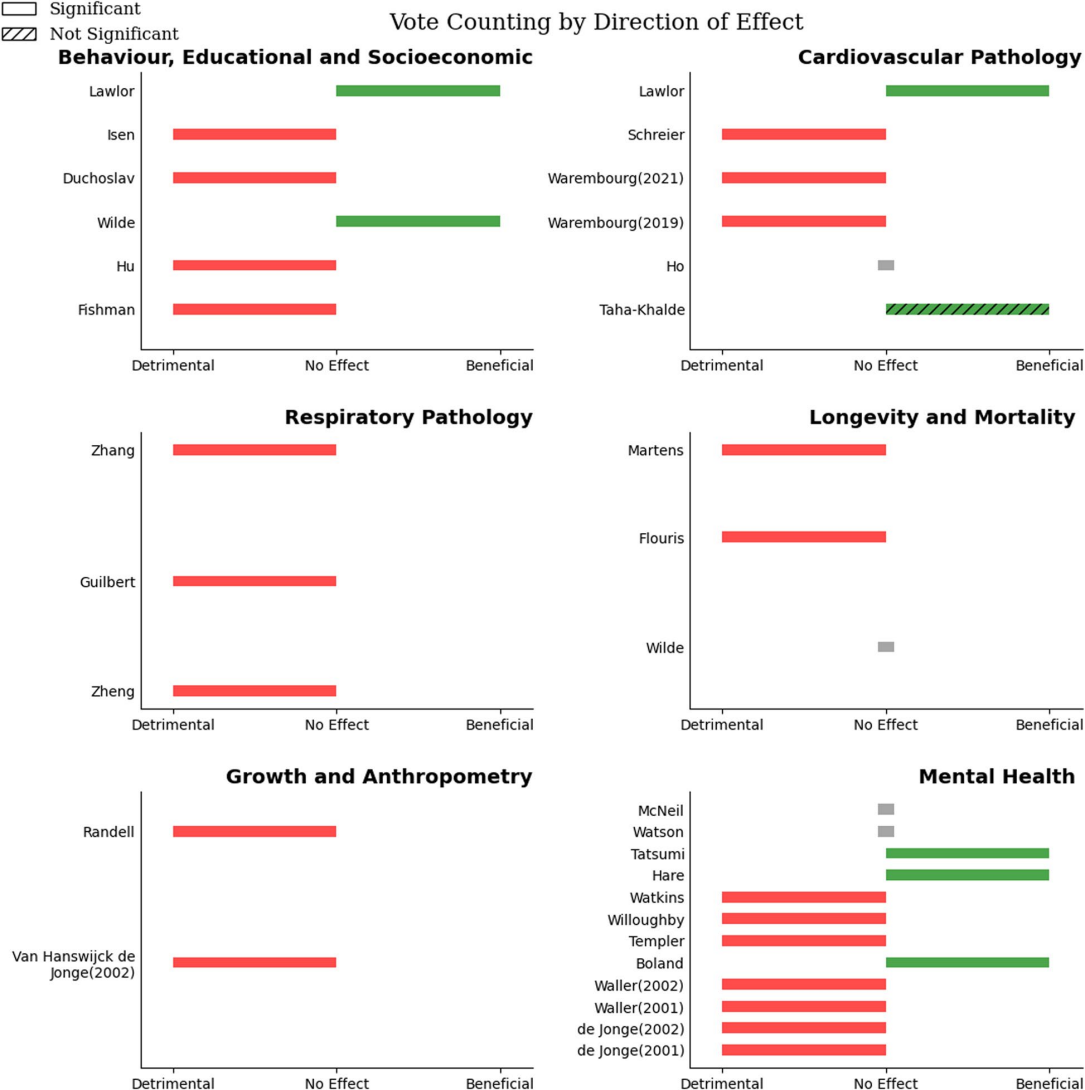
Figure showing vote counting across all outcome groups. No Effect = No direction of effect noted in study

A total of six studies reported on behaviour, educational and socioeconomic outcomes, which were detrimentally affected by increases in heat exposure (Fig. 4; Table 1), although the quality of the evidence was *very low*. End-points were not uniform, but included earnings, completion of secondary school or higher education, number of years of schooling, and gamified cooperation-rates in a public-goods game (where test scores represent achieving maximal public benefit in hypothetical situations).

**Table 1﻿:** Summary table-behaviour, educational and socioeconomic (Grade of evidence: *Very low*)

First Author	Year	Title	Study Type	Country	Population/Sample	Time Period of Birth	Measure of Heat Exposure	Time Period of Exposure	Outcomes	Risk of Bias	Summary of Results
Fishman [22]	2019	Long-term impacts of exposure to high temperatures on human capital and economic productivity	Cross-Sectional Analytical	Ecuador	*N* = 1 million.	1950–1980	Mean Temperature	9 Months before birth, and 9 months after birth	Earnings, Completing Secondary Education, Completing Higher Education,	**High**	For every 1℃ increase in mean temperature in 9 months before birth, there was a 0.7–1.2% decrease in earnings (*P* < 0.05). Highest impacts were noted above 28 °C. Higher losses in income were noted in females. Rates of completing secondary education was found to be reduced by 0.2% per 1℃ (*P* = 0.05). A 0.5% reduction in females completing higher education was also noted (*P* < 0.05). Other findings were non-significant.
Hu [23]	2019	Too hot to handle: The effects of high temperatures during pregnancy on adult welfare outcomes	Cross-Sectional Analytical	China	*N* = 9024	1950–1994	Days of Temperature > 85*F	Entire pregnancy	Mean years of schooling, Illiteracy, Standardised word-test score, mean height,	**High**	Analysis was conducted primarily on rural areas due to hypothesis testing, but the results in urban areas were noted to be non-significant. Per additional day of high temperature (> 85 F) exposure during the whole duration of pregnancy: the mean years of schooling was lowered by 0.02(coefficient = − 0.0157, SE=(0.009) *P* = 0.07); illiteracy was increased by 0.18% (coefficient = 0.0018, SE=(0.0009); *P* < 0.05); a standardized word-test score was 0.48% lower (coefficient = − 0.0048 SE = 0.0022; *P* < 0.05). When analysing the effect of timing of heat exposure, second trimester exposures had the largest impacts on educational outcomes, and the findings in the third trimester were non-significant.
Wilde [24]	2017	The effect of ambient temperature shocks during conception and early pregnancy on later life outcomes	Cross-Sectional Analytical	Burkina Faso, Cameroon, Guinea, Malawi, Rwanda and Uganda	*N* = 3,475,680	Unclear, census data(1976–2008)	Mean Monthly Temperature	Entire Pregnancy	Years of schooling(imputed based on education attained), Literacy, Disability, Mortality	**High**	Higher temperatures during pregnancy accounted for increased number of years of school completed, and increased rates of literacy. The highest effect sizes were noted at the time of conception and in the first trimester. Per 1 ℃ increase in mean temperature, years of schooling were increased by 0.0317(SE: 0.0133, *P* < 0.05) years, increases literacy rates by 0.00355 (SE:0.00104, *p* < 0.05).
Duchoslav [25]	2017	Prenatal Temperature Shocks Reduce Cooperation: Evidence from Public Goods Games in Uganda	Cohort	Uganda	*N* = 531.	Not Mentioned( cross-sectional analysis on 2014/07/01)	Mean Temperature	Entire pregnancy	Cooperation in Public Goods Game	**High**	Cooperation scores in a public-goods game were decreased by 20%, for every 1 °C increase in temperature exposure throughout gestation. The effect size was greatest in the first trimester, with second and third trimester effects not statistically significant
Isen [26]	2017	Relationship between season of birth, temperature exposure, and later life wellbeing	Cross-Sectional Analytical	United States	> 12 million	1969–1977	Additional Day > 32*C	Entire pregnancy and first year of life, including analysis on each trimester	Mean Annual Earnings	**High**	Decline in adult earnings from increasing temperature exposure throughout pregnancy. The effect was significant at temperatures above 28.1 °C. For each additional day of temperature > 32 °C during pregnancy or in the first year of life, there was noted to be a $30 decline in mean annual earnings at ages 29–31 (0.1% effect size). The largest effect size was noted in the first trimester, with a $55.735(SE: 15.425, *P* < 0.01) decrease in earnings per day > 32 °C.
Lawlor [27]	2004	Temperature at birth, coronary heart disease, and insulin resistance: cross sectional analyses of the British women	Cohort	United Kingdom	*N* = 4286	Unclear	Mean Monthly Temperature	Entire Pregnancy (First, Second, Third Trimester, Birth)	CHD, Triglycerides, Insulin Resistance, Age at Completion of Full Time Education	High	The outcomes were only statistically significant for a sub-analysis of temperature at birth, grouped by IQR. Age at completion of full-time education was higher in the highest temperature group, at 15.8 (95% CI: 15.6,16.1, *P* = 0.04 for trend).

Two large studies reported a detrimental effect of heat exposure on adult income, with the greatest effect noted in first trimester exposure. These studies noted a reduction in earnings of up to 1·2% per 1 °C increase in temperature, with greater effects in females [22], and a decrease of $55.735 (standard error(SE): 15·425, *P* < 0·01) annual earnings at 29–31 years old, per day exposure > 32 °C [26]. Two studies reported worse educational outcomes, with the greatest effect noted in the second trimester [23]. Rates of completing secondary education were found to be reduced by 0·2% per 1 °C increase in temperature (*P* = 0·05) [22], illiteracy was increased by 0·18% (SE=(0·0009); *P* < 0·05) and mean years of schooling was lowered by 0.02 (SE=(0·009) *P* = 0·07) [23]. Two studies reported a beneficial effect of heat exposure on educational outcomes, although both studies suffered from significant methodological flaws, and effects were < 0·01% when effect estimates were noted [24, 27]. One small study reported lower cooperation rates by 20% (*P* < 0·01) in a public-goods game, with lower predicted public wellbeing [25]. 

The studies generally exhibited a dose-response effect with evidence for a critical threshold of effect of 28 °C in one study [22]. All studies were at a high risk of bias.

Six studies reported on cardiovascular pathology and risk factors thereof, which were detrimentally affected by increased exposure to heat (Fig. 4; Table 2), although measures and surrogates of this outcome were heterogenous. The quality of the evidence was very low, and the sample sizes were small. Outcomes included blood pressure, a composite cardiovascular disease indicator, and specific cardiovascular disease risk factors such as diabetes mellitus (type I), insulin resistance, waste circumference, and triglyceride levels.

**Table 2:** Summary of findings cardiovascular pathology (Grade of evidence: *Very low*)

First Author	Year	Title	Study Type	Country	Population/Sample	Time Period of Birth	Measure of Heat Exposure	Time Period of Exposure	Outcomes	Risk of Bias	Summary of Results
Taha-Khalde [28]	2021	Air pollution and meteorological conditions during gestation and type 1 diabetes in offspring	Case-Control	Israel	Cases = 362. Controls = 3512	Not mentioned	Mean Temperature	Entire pregnancy, First, Second, Third Trimester and Birth	Incidence of Type 1 Diabetes	**Low**	Odds of having Type 1 Diabetes in quartile 4 versus quartile 1 of daily mean temperature during gestation was 0.73 lower (95%CI = 0.48, 1.09, *P*-value not stated), however the findings were not statistically significant.
Ho [29]	2015	Early-Life Environmental Exposures and Height, Hypertension, and Cardiovascular Risk Factors Among Older Adults in India	Cross-Sectional Analytical	India	*N* = 1036	1913–1962	Minimum Temperature Shock (Temp. >90th Percentile for month)	Entire Pregnancy, First, Second and Third Trimester	Height, Waist Circumference, Hypertension, Composite Cardiovascular disease indicator	**High**	Non-significant results for temperature shock (> 90th centile for the month) on height, hypertension, waist circumference and a composite cardiovascular disease indicator. Direction of effect not stated.
Warembourg [30]	2019	Early-Life Environmental Exposures and Blood Pressure in Children	Cohort	Crete, Lithuania, Spain, France, United Kingdom, Norway	*N* = 1,277	Unclear, 1999–2010	Mean Temperature	Entire pregnancy	SBP, DBP	**High**	Systolic blood pressure was associated with higher temperature exposure during pregnancy, where a 1.6 mm Hg increase was noted per IQR increase (95%CI = 0.2, 2.9, *P* = 0.024)
Warembourg [31]	2021	Urban environment during early-life and blood pressure in young children	Cohort	France, Greece, Spain and United Kingdom	*N* = 4,279	1999–2010	IQR Mean Temperature	Entire pregnancy	SBP, DBP	**High**	A change in BP per IQR mean temperature over the whole duration of pregnancy was noted as an increase in SBP of 1.2mmHg (95% CI =-0.0; 2.5, *p*-value not stated) and an increase of 0.6mmHg in DBP (95%CI = -0.6, 1.7, *p*-value not stated). Although this was a positive direction of effect, neither changes were statistically significant.
Schreier [32]	2013	Seasonality and ambient temperature at time of conception in term-born individuals – influences on cardiovascular disease and obesity in adult life	Cohort	Finland	*N* = 11,237	1934–1944	Mean Temperature	Conception	Hypertension, Coronary Heart Disease, Cerebrovascular Disease, BMI	**High**	With increasing temperature exposure at conception, a statistically significant increase in hypertension rates (*P* = 0.04) in women, and an increase in coronary heart disease was noted (*P* = 0.08). Men were noted to have a statistically significant decrease in BMI (*p* = 0.04) with increasing temperature exposure. Other outcomes were not statistically significant, and were not reported on.
Lawlor [27]	2004	Temperature at birth, coronary heart disease, and insulin resistance: cross sectional analyses of the British women	Cohort	United Kingdom	*N* = 4286 women	Unclear	Mean Monthly Temperature	Entire Pregnancy (First, Second, Third Trimester, Birth)	CHD, Triglycerides, Insulin Resistance, Age at Completion of Full Time Education	**High**	The outcomes were only statistically significant for a sub-analysis of temperature at birth, grouped by IQR. The incidence of CHD was lowest in the highest temperature group (*p* = 0.03 for trend), triglycerides were lowest in the highest temperature group (*p* = 0.06), and insulin Resistance was lowest in the highest temperature group (*p* = 0.04). The OR was only reported for CHD, and comparing the lowest temperature group to the highest three, it was noted to be 1.19 (95%CI: 0.95,1.48, *P*-value not stated) and thus although showing a negative direction of effect, it crossed the no effect line.

Three studies found a detrimental effect of heat exposure on hypertension rates, and increased blood pressure [31], with a maximum of 1·6 mm Hg increase noted per interquartile range (IQR) increase (95% Confidence interval (CI) = 0·2, 2·9, *P* = 0·024) in children [30], with increased effects on women in the largest study (*N* = 11,237) [32]. Another study found increasing heat exposure at conception was detrimentally associated with an increase in coronary heart disease (*P* = 0·08) [32], although one of the smaller studies (*N* = 4286) found a beneficial effect of heat exposure at birth on diverse cardiovascular outcomes, including coronary heart disease (*P* = 0·03 for trend), triglyceride levels (*P* = 0·06 for trend) and insulin resistance (*P* = 0·04 for trend) [27]. One study found lower odds of type I diabetes mellitus with increasing heat exposure, with odds ratio (OR) = 0·73 (95%CI = 0·48, 1·09, *P*-value not stated) [28]. Another study did not detect statistically significant relationships between heat exposure and hypertension or a composite cardiovascular disease indicator, but did not provide effect estimates [29]. Five studies were at a high risk of bias [27, 29–32], with only one case-control study at a low risk of bias [28]. 

Respiratory pathology was reported by three studies, assessing different outcomes. Outcomes were detrimentally associated with increasing heat (Fig. 4; Table 3), however the quality of the evidence was *low*. The outcomes were primarily measured in infants and children, with no studies on adult outcomes. The largest study (*N* = 1681) found that increasing heat exposure increased the odds of having childhood asthma [33], and another small study (*N* = 343) noted worsened lung function with increasing heat exposure [34].

**Table 3:** Summary of findings of respiratory pathology (Grade of evidence: *low*)

First Author	Year	Title	Study Type	Country	Population/Sample	Time Period of Birth	Measure of Heat Exposure	Time Period of Exposure	Outcomes	Risk of Bias	Summary of Results
Guilbert [34]	2023	Association of Prenatal and Postnatal Exposures to Warm or Cold With Temperatures With Lung Function in Young Infants	Cohort	France	*N* = 343	July 2014- July 2017	Daily Minimum, Mean, Maximum and Variability	Entire Pregnancy and First Four Weeks after Delivery. ?35–40 weeks excluded.	mean minute ventilation, tidal volume, respiratory rate (RR), and time to peak tidal expiratory flow to total expiratory time (tPTEF/tE) ratio	**High**	A decrease in FRC was noted with increasing temperature exposure (95th vs. 50th percentile) in the second and third trimester in female infants. This equated to a decrease of 39.7 ml (95%CI, -68.6 to -10.7mL, *p*-value not stated) with an associated increase in RR by 28.0/min (95% CI 4.2–51.9/min, *p*-value not stated). Of note, similar findings were noted with exposure to cold(5th vs. 50th percentile) in female infants. Decreased tidal volume by 23.8mL (95% CI, -42.4 to -1.3mL). RR increased by 45.5/min (95% CI 10.1–81.0/min).
Zhang [33]	2022	Maternal apparent temperature during pregnancy on the risk of offspring asthma and wheezing: effect, critical window, and modifiers	Case-Control	China	*N* = 1681(cases = 236, controls = 1445)	December 2018-March 2019	Apparent Temperature(Mean)	Entire Pregnancy, First, Second, Third Trimester	Self-Reported Doctor-Diagnosed Asthma or Wheezing	**Low**	Increasing heat exposure was found to have a positive association in first trimester, but not statistically significant. The association in the second trimester was not significant. Heat exposure in the third trimester was found to be significantly associated with risk of asthma at temperatures of > 24.65 °C, with a maximum OR of 1.926(95% CI 1.208–3.070, *p*-value not stated) at AT 31.10 °C. Of note, cold exposure in the first and third trimesters were also noted to be associated with an increased risk of asthma.
Zheng [35]	2021	Preconceptional and prenatal exposure to diurnal temperature variation increases the risk of childhood pneumonia	Case-Control	China	N cases = 699 (WHO diagnostic criteria for pneumonia). N controls = 811. Children < 14 years	Unclear(2003–2019)	Maximum Temperature minus Minimum Temperature	Entire Pregnancy(First, Second, Third Trimester)	Diagnosis of Childhood Pneumonia	**High**	An increase in the odds of having childhood pneumonia were noted, with a maximum OR = 1.85 (95%CI = 1.24, 2.76) in the third trimester, with OR = 1.63 (1.32, 2.00), and OR = 1.43 (95%CI = 1.12, 1.81) in the second and third trimester respectively. The effect was increased in males compared to females.

An additional study noted increased odds of childhood pneumonia with increasing diurnal temperature variation (DTV) in pregnancy, with a maximum OR = 1·85 (95%CI = 1·24, 2·76) in the third trimester [35].

Exposure in the third trimester had the greatest effect across all three studies [33–35]. Females showed an increased susceptibility to heat exposure’s effects on lung function, but males were more susceptible to heat’s effect on childhood pneumonia. There was a critical threshold noted in the asthma study of 24·6 °C, with a dose-response effect. The asthma study was assessed as low risk of bias, however the other studies were at high risk.

Growth and anthropometry was reported on by two studies, with differing outcomes, although in both, heat exposure was associated with detrimental, although heterogenous, outcomes (Fig. 4; Table 4). The overall quality of the evidence was *very low*. One study found a positive association with heat exposure and increased body mass index (BMI), *r* = 0·22 (*P* < 0·05) in the third trimester with greater effects noted in females and in African-Americans [36]. Another large study (*N* = 23 026) found increased odds of stunting (OR = 1·28, 95%CI = not stated, *p* < 0·001) with a negative correlation with height noted (*r*=-0·083 *P* < 0·01) [37]. Effects were greatest in the first and third trimester. Both studies were at a high risk of bias.

**Table 4:** Summary of findings growth and anthropometry (Grade of evidence: *Very low*)

First Author	Year	Title	Study Type	Country	Population/Sample	Time Period of Birth	Measure of Heat Exposure	Time Period of Exposure	Outcomes	Risk of Bias	Summary of Results
van Hanswijck de Jonge [36]	2002	Environmental temperature during gestation and body mass index in adolescence: new etiologic clues?	Cross-Sectional Analytical	United States	*N* = 578	Not Reported	Mean Temperature	Entire Pregnancy, Birth, First, Second and Third Trimester	BMI Z-scores, BMI Categorical Variable	**Low**	The correlation was only statistically significant within a sub-population analysis of African-American females. A weakly positive correlation was observed, with a coefficient of 0.21 (*P* < 0.05) in the second trimester and 0.22 (*P* < 0.05) in the third trimester. Other associations were neither consistent in their direction of effect, nor were they statistically significant. An analysis was conducted on BMI and temperature at birth as categorical variables (> 85th centile, > mean temperature respectively). This was not statistically significant for males, but females had a RR of 2.30 (95%CI = 1.15–4.60, *P* = 0.02).When further separated, African American females (RR = 2.51, 95%CI = 1.07, 5.91, *p* = 0.04) had a higher RR than white females, although this was not statistically significant. (RR = 1.94, 95% CI = 0.61, 6.14 *P* = NS).
Randell [37]	2020	Stunted from the start: Early life weather conditions and child undernutrition in Ethiopia	Cohort	Ethiopia	*N* = 23,026	1995–2015	Mean Temperature	Entire Pregnancy, First, Second and Third Trimester	HFA Z-score, Odds of Stunting	**High**	A 1 °C increase in mean temperature throughout the whole of pregnancy increases the odds of severe stunting by 28% (OR:1.28, *p* < 0.001; 95%CI = to calculate).Similarly, a 1 °C increase in mean temperature was associated with an increased risk of stunting of 16% (OR: 1.16, *P* < 0.01, 95% CI = to calculate). Height for age Z-score coefficient was negatively correlated with increasing temperature, with a regression-coefficient of -0.083 (*p* < 0.01). Severe stunting increases with increasing mean temperature in each trimester, with that effect highest in the 1st trimester. Stunting in general also increases with increasing temperature, but was highest in the 3rd trimester (*P* < 0.1), and although increased, were not statistically significant in other trimesters.

Mental health was reported on by 12 studies. Increasing heat exposure generally had a detrimental association with mental health outcomes (Fig. 4; Table 5), although these were heterogenous. The overall quality of the evidence was *very low*. Five studies reported on schizophrenia rates, with only one study showing a strongly positive association of heat exposure at conception with schizophrenia rates (*r* = 0·50, *p* < 0·025) [38]. Another study noted the same effect with increasing heat in the summer before birth, however this was not statistically significant [39]. The third study reported no association of this outcome [40], with another small study (*N* = 2985) showing a negative correlation with temperatures at birth, without reporting on heat exposure during other periods of gestation [41]. The fifth study failed to report direction of effect, but noted non-significant findings [42]. Six studies reported on eating disorders, with all six showing a detrimental effect with increasing heat exposure. Of the three studies on clinical anorexia nervosa, one reported increasing rates of anorexia nervosa compared to other eating disorders (χ²= 4·48, *P* = 0·017) [43], another reported increasing rates of a restrictive-subtype (χ²= 3·18, *P* = 0·04) as well as reporting worse assessments of restrictive behaviours [44], which was supported by a third study in a different setting [45]. Three studies examined non-clinical settings, with some inconsistent effects. The first study showed a weak positive association with heat exposure, and drive for thinness (Spearman’s ⍴ = 0·46, *P* < 0·05) and bulimia scores (Spearman’s ⍴ = 0·25, *P* < 0·05) [46], which was supported by a replication study [47], and one other study [48]. The most significant and consistent effects noted in the third trimester, at birth, and in females [47, 48]. One study reported a beneficial effect of increased temperatures in the first trimester on rates of depression, however no other directions of effect were noted for other periods of exposure [49]. These studies were at a high risk of bias.

**Table 5:** Summary of findings mental health (Grade of evidence: *Very low*)

First Author	Year	Title	Study Type	Country	Population/Sample	Time Period of Birth	Measure of Heat Exposure	Time Period of Exposure	Outcomes	Risk of Bias	Summary of Results
van Hanswijck de Jonge [47]	2002	Influence of environmental temperatures during restation and at birth on eating characteristics in adolescence	Cohort	United States	*N* = 578	Not Reported	Mean Temperature	Entire Pregnancy, Birth	Drive for Thinness, Bulimia, Body Dissatisfaction (sub-scales of EDI)	**High**	Increasing temperature exposure was weakly associated with increased drive for thinness scores in the second trimester in females (Spearman’s ⍴ = 0.14, *P* < 0.05) and increasing bulimia scores in the third trimester in males (Spearman’s ⍴= 0.13, *P* < 0.05), but decreased bulimia scores in the first trimester (Spearman’s ⍴= -0.12, *P* < 0.05), and at birth in males (Spearman’s ⍴ = -0.12, *P* < 0.05). No significant effects were noted with body dissatisfaction.
van Hanswijck de Jonge [46]	2001	Environmental Temperature During Pregnancy and Eating Attitudes During Teenage Years: A Replication and Extension Study	Cross-Sectional Analytical	United Kingdom	*N* = 128	Not Reported	Mean Monthly Temperature	First, Second, Third Trimester and Birth	Drive for Thinness, Bulimia, Body Dissatisfaction (sub-scales of EDI)	**High**	An increased drive for thinness correlated with temperature at birth (Spearman’s ⍴ = 0.46, *P* < 0.05) in older males, but other outcomes were non-significant for older males and females. Younger females had an increased Drive for Thinness score with increasing temperature exposure in second trimester (Spearman’s ⍴ = 0.31, *P* < 0.05). Increasing temperature exposure in first trimester decreased bulimia scores (Spearman’s ⍴ = -0.30, *P* < 0.05), and in the third trimester increased scores (Spearman’s ⍴ = 0.25, *P* < 0.05) in younger females.
Waller [48]	2001	Early Environmental Influences on Restrictive Eating Pathology Among Nonclinical Females: The Role of Temperature at Birth	Cohort	United Kingdom	*N* = 117	Not Reported	Mean Monthly Temperature	First, Second, Third Trimester and Birth	Drive for Thinness, Bulimia, Body Dissatisfaction (sub-scales of EDI)	**High**	Individuals born in warmer vs. cooler months, increased scores for Drive for Thinness (*P* = 0.01) and Body Dissatisfaction (*P* = 0.03) were noted, however effects on Bulimia were non-significant. There was a positive association between temperature at birth and Drive for Thinness Score (Spearman’s ⍴ =0.22, *P* < 0.01). The relationship for Bulimia and Body Dissatisfaction was in a positive direction of effect but was non-significant.
Waller [44]	2002	Pattern of Birth in Adults with Anorexia Nervosa	Cohort	United Kingdom	*N* = 195 (Restrictive Sub-Type: *N* = 117, Binge-Purge: *N* = 78)	Not Reported	Mean Monthly Temperature, Mean Quarterly Temperature	Conception, Birth, Entire Pregnancy	Diagnosis, EAT-26 Score, BMI	**High**	The results were not significant for correlation of BMI, and Diagnosis of Restrictive vs. Binge-Purge sub-type when comparing temperature at month of conception(direction of effect not specified), but significantly increased diagnosis of restrictive sub-type when comparing warmest quarter vs. all others. Chi-squared Test [Yates’ continuity correction] = 3.18, df = 1, one-tailed *P* = 0.04. In addition, worse restrictive eating behaviours was correlated with temperature at conception in the restrictive sub-type group only (Spearman’s ⍴ = 0.40, *P* = < 0.01).
Boland [49]	2018	Uncovering exposures responsible for birth season – disease effects: a global study	Cohort	United States, South Korea, and Taiwan	*N* = 10.5 million	Not Reported	Minimum and Maximum Temperature (over a quarter)	First, Second, Third Trimester and Birth, Entire Pregnancy	133 Diseases, including ADHD and Depression	**High**	Depression was negatively correlated with temperature in the first trimester (high temperature, *R* = -0.645, 95% CI, -0.462 to -0.779, *p*-value not stated, reported significant; low temperature, *R* = 0.651, 95% CI, 0.446 to 0.790, *p*-value not stated, reported significant). No other associations or direction of effect were noted.
Templer [38]	1980	Confirmation of Relationship Between Temperature and the Conception and Birth of Schizophrenics	Cohort	United States	N = not reported	Not Reported	Mean Monthly Temperature	Conception, Birth	Schizophrenia	**High**	Schizophrenia birth rates were associated with lower temperatures at birth (*r* =- 0.48, *P* < 0.025) and Schizophrenia conception rates were associated with higher temperatures at conception (*r* = 0.50, *P* < 0.025).
Willoughby [45]	2001	Pattern of Birth in Anorexia Nervosa II: A Comparison of Early-Onset Cases in the Southern and Northern Hemispheres	Cohort	Australia, United Kingdom	*N* = 458	1971–1990	Mean Monthly Temperature	Birth, Conception	Anorexia (Restrictive vs. Binge-Purge Subtype)	**High**	Anorexia(restrictive-subtype) birth rates were associated with lower temperatures at birth (Chi-squared Test (Yate’s continuity correction) = 2.81, df = 1, *P* = 0.047)and Anorexia (restrictive-subtype) conception rates were associated with higher temperatures at conception (z = 0.079, *P* = 0.937), although this was not statistically significant. The authors contributed this to the correlation of low birth temperatures with high temperatures at conception.
Watkins [43]	2001	Pattern of Birth in Anorexia Nervosa I: Early-Onset Cases in the United Kingdom	Cohort	United Kingdom	*N* = 259 (Anorexia) *N* = 149 (other eating disorders)	Not Reported	Mean Monthly Temperature	Birth, Conception	Anorexia vs. Other Eating Disorder	**High**	Anorexia conception rates were associated with increasing temperature (z = 2.26; *P* = 0.024) when compared to other-eating disorders. Associated also noted with categorical approach [Chi-squared (Yate’s correction for continuity) = 4.48; df = 1; *P* = 0 0.017]
Hare [39]	1981	A relation between seasonal temperature and the birth rate of schizophrenicpatients	Case-Control	United Kingdom	Schizophrenia, *N* = 17,217; Affective Psychosis *N* = 14,004; Neurosis, *N* = 43,657; and Personality Disorder (*N* = 16,265)	1921–1955	Mean Quarterly Temperature	Birth	Schizophrenia, Affective Psychosis, Neuroses, Personality Disorder	**High**	NS for Neurosis, Personality Disorder, Affective Psychosis. The largest correlation was noted with schizophrenia birth rates in the second quarter increasing with lower temperatures over the first half of the year (*r* = -0.67, *p* < 0.001), which was consistent with other comparisons which suggested years with colder temperatures had an increased schizophrenia birth rate. Of note, the schizophrenia birth rates were higher with increasing temperatures at conception, however this failed to reach statistical significance (*r* = 0.16, *P* > 0.1)
Tatsumi [41]	2002	Season of birth in Japanese patients with schizophrenia	Case-Control	Japan	*N* = 2985	1920–1965	Mean Monthly Temperature	Birth	Schizophrenia	**High**	Significant negative correlation with temperature at birth and schizophrenia birth rates (Spearman’s ⍴ = -0.68, *P* = 0.015) in male patients, with opposite direction of effect in female population (Spearman’s ⍴ = 0.48, *P* = 0.12(NS)). They did not assess the temperature at conception to account for this. 14% decrease in expected schizophrenia rates in warmest 3 months (*P* = 0.001), 8% increase in schizophrenia rates in coldest 3 months was noted (*P* = 0.04).
Watson [40]	1984	Schizophrenic Birth Seasonality in Relation to the Incidence of Infectious Diseases and Temperature Extremes	Cohort	United States	*N* = 3246	1915–1978	Mean Monthly Temperature	Summer before birth	Schizophrenia	**High**	Results NS, no direction of effect reported.

Increasing heat exposure had a detrimental effect on longevity and mortality across various outcomes (Fig. 4; Table 6), although despite large sample sizes, the quality of the evidence was *low*. One study found a negative correlation of heat exposure with longevity (*r*=-0·667, *P* < 0·001), with a greater effect on females [50]. A second study showed a detrimental effect on telomere length, as a predicter of longevity, with the greatest effect towards the end of gestation (3·29% shorter TL, 95%CI = − 4·67, − 1·88, per 1 C increase above 95th centile) [51]. Conversely, a third study noted no correlation with mortality [24]. All but the study on telomere length [51] were at a high risk of bias.

**Table 6:** Summary of findings longevity and mortality (Grade of evidence: *low*)

First Author	Year	Title	Study Type	Country	Population/Sample	Time Period of Birth	Measure of Heat Exposure	Time Period of Exposure	Outcomes	Risk of Bias	Summary of Results
Wilde [24]	2017	The effect of ambient temperature shocks during conception and early pregnancy on later life outcomes	Cross-Sectional Analytical	Burkina Faso, Cameroon, Guinea, Malawi, Rwanda and Uganda	*N* = 3,475,680	Unclear, census data (1976–2008)	Mean Monthly Temperature	Lagm-9 (secondary review of all months)	Adult Disability and Child Mortality	**High**	The results as they pertained to infant mortality had a small negative effect, but were not statistically significant . The regression coefficient of infant mortality is -0.0006, essentially no effect (SE and *P*-value not reported).
Flouris [50]	2009	Effect of seasonal programming on fetal development and longevity: links with environmental temperature	Cross-Sectional Analytical	Greece	1) Birth outcomes *N* = 516,874) Longevity analysis *N* = 554,101	1999–2003 for BW/Prem analysis, Deaths between 1999–2003 (unclear date of birth)	Mean Temperature	Month of Birth, Year of Birth	Longevity, Birth Weight, Gestational Age at Birth	**High**	Higher temperatures in the year of birth were correlated with a shorter lifespan *r*=-0.667 (*P* < 0.001). This effect was more pronounced in females. Males: *r*=-0.623 (*P* < 0.001); females: *r*=-0.701 (*P* < 0.001).
Martens [51]	2019	Early Biological Aging and Fetal Exposure to High and Low Ambient Temperature: A Birth Cohort Study	Cohort	Belgium	*N* = 1,103	February 2010 and December 2016	Mean Temperature	Whole of pregnancy + weekly analysis	Telomere Length at Birth	**Low**	Shorter TL with increasing temperature exposure in-utero with a statistically significant effect noted in weeks 14–36 gestation. The effect was highest at week 36, with a 3.29% shorter TL (95%CI = − 4.67, − 1.88, *P*-value not stated) per 1 C increase above 95th centile. Whole of pregnancy showed a 1.49% decrease (95%CI=-2.7, − 0.90) per 1 C increase above 95th centile.

## Discussion

This study establishes significant patterns of effects amongst the outcomes reviewed, with increasing heat exposure being associated with an overall detrimental effect on multiple, diverse, long-term outcomes. These effects are likely to increase with rising temperatures, however modelling this is beyond the scope of this review.

The most notable detrimental outcomes are related to neurodevelopmental pathways, with behavioural, educational, socioeconomic and mental health outcomes consistently associated with increasing heat exposure, in addition to having the greatest body of literature to support this. Importantly, other systems such as the respiratory and cardiovascular systems also suggest harmful effects of heat exposure, culminating in detrimental associations with longevity and mortality. Some studies illustrated a possible beneficial effect in some disease-processes, such as coronary heart disease and depression showing the potential for shifting disease profiles with rising temperatures.

The detrimental effects of heat exposure became more significant with increasing temperatures, with many studies describing increasing effects beyond critical thresholds which, although varied across studies, suggest that there is a limit of heat adaption strategies, both biological and behavioural [52, 53].

In addition, the effect of increasing heat exposure was associated with worse outcomes in already marginalised communities, such as women [22, 32, 34, 36, 44, 47, 48, 50] and certain ethnic groups (African-Americans) [46]. The reasons for sub-population vulnerabilities are unclear and likely complex. In the case of female foetuses being more susceptible to changes in the in-utero environment, it is possible that there is a ‘survivorship bias’. This would occur if women with harmful exposure lose male infants during pregnancy at a higher rate, and thus the surviving female infants appear more at risk. However, despite an increased risk of early pregnancy loss, there are no studies that have assessed this differential vulnerability. This still has the effect of potentially increasing the burden of disease on an already marginalised group.

In the case of certain population groups being more at risk, it is likely that both physiological differences in vulnerability as well as socio-economic effect-modifiers exist to explain these differences, however, the included literature lacks sufficient evidence to assess this. The vulnerabilities of different populations to the long-term effects of heat exposure in-utero likely contributes to the unequal impacts of climate change that have already been established [54], and will be an important contributor to inequality with future increases in temperature. Further research in this area is critical to inform targeted redistributive interventions.

Although the associations may be clear, establishing causality is fraught with difficulty, with no consensus on an infallible approach [55–57]. However, it is prudent to highlight supporting evidence in this review.

The hypothesis that the in-utero environment had significant long-term impacts on the foetus was first suggested by Barker, in the context of maternal nutrition and cardiovascular disease [11]. Further studies supported this hypothesis, and expanded on the effects the in-utero environment has on the foetus and its long-term wellbeing [58]. Long-term heat exposure may also be associated with changes in nutritional availability [11], and is likely one of many complex but important environmental exposures in-utero.

Maternal comorbidities, associated with increasing heat exposure such as hypertensive disorders of pregnancy and gestational diabetes mellitus, are known to negatively affect the foetus in the long-term [59, 60]. These comorbidities may be part of the long-term pathogenicity of heat exposure, through short-term exposure-outcome pathways. Placental dysfunction is central to the pathology of pre-eclampsia, and is a significant cause for foetal pathology [61, 62]. The placenta is not auto-regulated and is therefore acutely affected by changes to blood volume, heart rate and blood pressure, culminating in cardiac output as it is delivered to the placenta as an end-organ with resultant negative effects on the foetus [63]. Heat-acclimatisation mechanisms are hypothesized to affect this delicate balance [52, 64], with observational studies supporting this [64]. It has been suggested that heat exposure’s increase in inflammation is a possible causative mechanism for pre-term birth [5, 52], but inflammation has numerous additional effects on the immune system and could prove an insult to the mother and developing foetus [62, 65]. These effects may only manifest in the long-term.

Heat was one of the earliest described teratogens [66], with significant effects on neurodevelopment noted in animal models in keeping with the observed associations of this review [67]. Biological organisms are extremely dependent on heat as a trigger for various processes. Plants and animals undergo significant change in response to the seasons, which are often guided by fluctuations in temperature. These changes are often mediated by epigenetic mechanisms, allowing the modification and modulation of gene expression [68, 69].

**Fig. 5:**
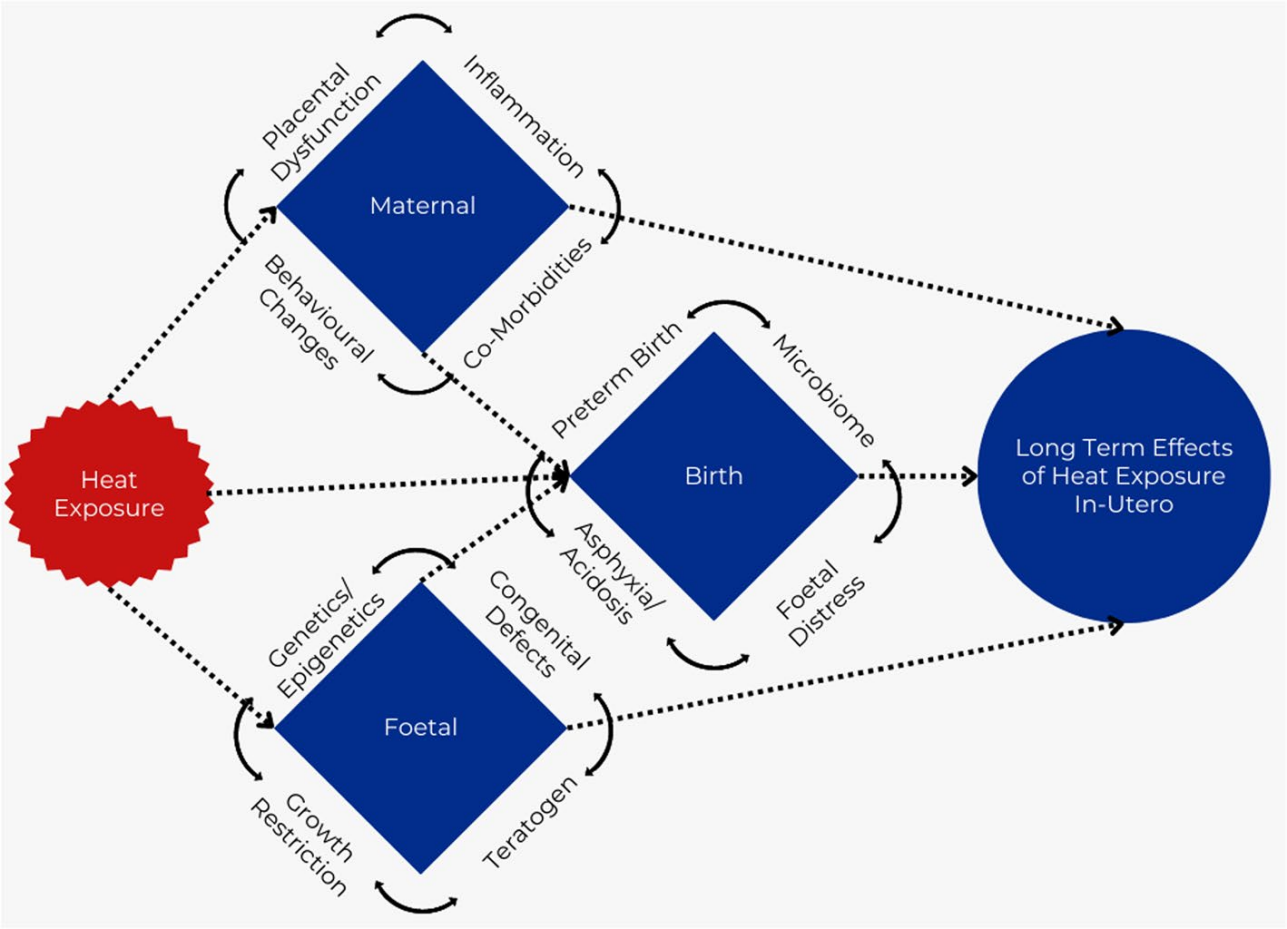
Causal pathways

Thus, from an evolutionary perspective, DNA, is sensitive to changes in temperature. The mechanism of this sensitivity has been shown to be primarily epigenetic in nature [69]. Increasing heat results in modifications to histone deacetylation and DNA methylation [69]. This is required to provide fast-acting adaptions to acute stressors, but can have long-term effects too [70]. Thus, it is likely that humans are sensitive to changes in temperature, which can alter epigenetic modifications, and thus our exposome. This sensitivity, may have provided a survival benefit in times of increasing heat, or it may simply be a vestigial function which provides no survival benefit, and may in fact have detrimental effects [71]. Epigenetic changes have been shown to have significant effects on metabolic diseases and risk profiles, and an in-depth review is provided by Wu et al. [72]. The exact processes and genes involved would be an area requiring further research, where similar research exists on the effects of nutrition on exact epigenetic pathways [73]. An important pattern requiring further research involves the effect heat may have on neurodevelopment [67, 74]. The above pathways provide additional mechanisms for the long-term lag between exposure-outcome pathways. In addition, acute heat exposure at the time of birth has been associated with various possibly pathogenic mechanisms such as preterm birth [5], low APGAR scores [75] and foetal distress [76], as well as a possible effect on the maternal microbiome and the seeding thereof to the neonate [10, 64, 77, 78]. These effects, can all provide plausible causes for the long-term outcomes observed through short-term insults. The interplay of these, and additional factors is highlighted in Fig. 5 [79]. Importantly, the periods of vulnerability are likely different for these various pathways, but specific outcomes may have multiple periods of vulnerability through different pathways.

The outcomes associated with increasing heat exposure highlight the health, social, and economic cost of global warming, establishing current estimates and future predictions for this are beyond the scope of this research but would provide a valuable area for future research. This would entail estimating disease-burden due to climate change through attribution studies. Traditional health impact studies conflate adverse outcomes from natural variations in climate (‘noise’) with adverse outcomes from anthropogenic climate change. However, not every climate-related adverse outcome is the result of anthropogenic climate change, and these effects are likely different in vulnerable populations. This highlights the benefit of studying and implementing effective heat adaptation strategies in areas where the greatest effect is likely to be observed, and where the greatest impacts in lessening the economic and human impact of global warming are possible [80, 81].

### Limitations

The difficulty in assessing the data is compounded by the heterogenous measures of heat exposure. No studies used widely accepted heat exposure indices that consider important environmental modifying factors like humidity and windspeed [82, 83]. In addition, effect modifiers, heat acclimatisation and adaptation strategies were seldom considered [84–86]. It may be prudent for future studies to consider the measure of ionizing radiation exposure as an analogous environmental exposure, where different measures exist for the intensity, total quantity (a function of duration of exposure) and biologically-adjusted quantity absorbed [87]. Differing time-periods of exposure made it difficult to evaluate specific periods of sensitivity, which are likely different for various outcomes, depending on critical periods of development.

Despite consistency across different contexts in this review, the analysis of the distribution of the included studies highlights the unequal weight of studies towards relatively cooler climates, in regions with higher socioeconomic levels and likely greater heat adaptation uptake, and must therefore be interpreted in this context. It is possible that myriad factors that differ geographically, including physiological and socio-economic differences, will influence the effects of heat, and thus there is likely no underlying universal truth to associations and effect estimates.

Quantifying, describing and comparing the effect size across studies was rendered more difficult due to heterogenous statistical analyses.

Although some studies adjusted for possible confounding variables, not all reported on this, with the effects of seasonal, foetal, and maternal biological factors that may not lie on the causal pathway seldom considered [3, 5, 9, 88–92].

Data extraction and assessment of risk of bias was not uniformly undertaken in duplicate due to resource constraints, which may predispose to extraction errors or bias. The high risk of bias of included studies, limits the utility of the overall assessment of effects and suggestions for further action. In addition, publication bias is likely skewing the results towards statistically significant detrimental results, with studies with smaller sample sizes not necessarily showing wider distribution of findings as would be expected.

## Conclusions

Climate change, and in particular, global warming, is a significant emerging global public health threat, with far reaching, and disproportionate effects on the most vulnerable populations. The effects of increasing heat exposure in utero are associated with, and possibly causal in, wide-ranging long-term impacts on socioeconomic and health outcomes with a significant cost associated with increasing global temperatures. This association is as a result of a complex interplay of factors, including through direct and indirect effects on the mother and foetus. Further research is urgently required to elicit biological pathways, and targets for intervention as well as predicting future disease-burden and economic impacts through attribution studies.

### Supplementary Information

**Additional file 1.** Full data extraction table including risk of bias and GRADE assessments.

**Additional file 2.** Search terms for Medline(PubMed) and Web of Science.

**Additional file 3.** Author list for Climate and Heat-Health Study Group. Individual JBI risk of bias assessment forms, and excluded studies metadata from EPPI reviewer available on request.

## Data Availability

This study was a review of publicly available information data, with references to data sources made in the reference list.

## Abbreviations

AT: Apparent Temperature
BMI: Body Mass Index
BP: Blood Pressure
CHD: Coronary Heart Disease
CI: Confidence Interval
DBP: Diastolic Blood Pressure
EDI: Eating Disorder Inventory
FRC: Functional Residual Capacity
IQR: Interquartile Range
NS: Non-Significant
OR: Odds Ratio
RR: Respiratory Rate
SBP: Systolic Blood Pressure
SE: Standard Error
TL: Telomere Length
WHO: World Health Organisation
