# Supplementary material for: Impacts of heat exposure in utero on long-term health and social outcomes: a systematic review
Source: BMC Pregnancy Childbirth. 2024 May 4;24:344. doi: 10.1186/s12884-024-06512-0 (PMC11069224; doi:10.1186/s12884-024-06512-0)
Supplement: Supplementary file 2 — Additional file 2. Search terms for Medline(PubMed) and Web of Science. [file 12884_2024_6512_MOESM2_ESM.docx]

# Search strategy on MEDLINE (Pubmed) and Web of Science, as used in mapping review

**Search covers terms for:**

1. **The population (those affected by heat: no 1 below) AND**
2. **Intervention (No. 2a: climate change OR 2b Media OR 2c cooling OR 2d Health promotion)**
3. **NOT 3 (genetics)**
4. **Search for the Population (those with heat-related conditions)**

(((“ambient temperature”[title/abstract] OR “heat strain”[title/abstract] OR “heat exposure”[title/abstract] OR “heat stress”[title/abstract] OR Heating[mesh] OR “Heat Stress Disorders”[MESH] OR “hot temperature*”[title/abstract] OR “extreme heat”[MESH] OR “Heat stroke”[MESH] OR “heatstroke”[title/abstract] OR “heat index”[title/abstract] OR “heat episode”[title/abstract] OR “Heat Stress Disorders”[MESH] OR “heat event”[title/abstract] OR “Body temperature”[Mesh:noexp] OR “extreme temperature*”[title/abstract] OR “summer temperature*”[title/abstract] OR “Heat Exhaustion”[mesh] OR “summer weather”[title/abstract] OR “summer temperature*”[title/abstract] OR “heat wave”[title/abstract] OR “heatwave”[title/abstract] OR “indoor temperature”[title/abstract] OR “global temperature*”[title/abstract]))) NOT ((("Animals"[Mesh] NOT ("Animals"[Mesh] AND "Humans"[Mesh])))) NOT (“Chemicals and Drugs Category”[MeSH]) NOT ((("Plants"[Mesh] NOT ("Plants"[Mesh] AND "Humans"[Mesh]))))

**AND**

1. **Search strategy for interventions**
   1. **locating Climate change articles in Pubmed**

((“global warming”[Title/Abstract] OR “global warming”[MESH] OR climatic*[Title/Abstract] OR “climate change"[Title/Abstract] OR “climate change”[MESH] OR “Desert Climate”[mesh] OR “El Nino-Southern Oscillation”[Mesh] OR Microclimate[mesh] OR “Tropical Climate”[mesh]))

- 1. **Search for media (a validated search for social media)**

media[title/abstract] OR communication*[title/abstract] OR audiovisual[title/abstract] OR helpline*[title/abstract] OR hotline*[title/abstract] OR telecommunication*[title/abstract] OR educat*[title/abstract] OR radio[title/abstract] OR television[title/abstract] OR TV[title/abstract] OR internet[title/abstract] OR campaign*[title/abstract] OR advert*[title/abstract] OR print*[title/abstract] OR “information campaign”[title/abstract] OR telemedicine[title/abstract] OR telehealth[title/abstract] OR telepharmac* OR e-health[title/abstract] OR ehealth[title/abstract] OR e-pharmac*[title/abstract] OR “social media”[mesh] OR “social media”[title/abstract] OR “social network*”[title/abstract] OR Twitter[title/abstract] OR Facebook[title/abstract] OR Linkedin[title/abstract] OR Pinterest[title/abstract] OR YouTube[title/abstract] OR "daily motion"[title/abstract] OR Yelp[title/abstract] OR Foursquare[title/abstract] OR "Google Circles"[title/abstract] OR Qype[title/abstract] OR Ello[title/abstract] OR Instagram[title/abstract] OR "Pip.io"[title/abstract] OR "Google Buzz"[title/abstract] OR Orkut[title/abstract] OR Tumblr[title/abstract] OR onesocialweb[title/abstract] OR asmallworld[title/abstract] OR bebo[title/abstract] OR myspace[title/abstract] OR folkdirect[title/abstract] OR virb[title/abstract] OR Friendster[title/abstract] OR storyofmylife[title/abstract]

- 1. **Search for cooling**

cooling[title/abstract] OR “Air condition*”[title/abstract] OR “Air-condition*”[title/abstract] OR climatization[title/abstract] OR climatisation[title/abstract] OR ventilation[title/abstract] OR Fan[title/abstract] OR fans[title/abstract]

- 1. **Search for health promotion and risk**

adaptation[title/abstract] OR adapting[title/abstract] OR response[title/abstract] OR alert[title/abstract] OR implement*[title/abstract] OR awareness[title/abstract] OR strategy*[title/abstract] OR strategies[title/abstract] OR ”risk management”[title/abstract] OR ”risk management”[mesh] OR “emergency management”[title/abstract] OR “preparedness”[title/abstract] OR “disaster management”[title/abstract] OR warning*[title/abstract] OR “health promotion”[MeSH] OR “Health education”[MeSH] OR “communication campaign*”[Title/abstract] OR guideline*[Title/Abstract] OR recommendation*[Title/Abstract] OR “Health Services for the Aged”[mesh] OR “health facility planning”[MESH] OR “Public health surveillance”[MeSH] OR “home intervention”[title/abstract] OR “home care”[title/abstract] OR homecare[title/abstract] OR “home care services”[MESH] OR “patient care planning”[MESH] OR “Comprehensive Health Care”[mesh] OR outreach[title/abstract] OR “Patient care team”[MESH] OR multidisciplinary[title/abstract] OR “home visit”[title/abstract] OR “home assessment”[title/abstract] OR “patient management”[title/abstract] OR “social support”[MESH] OR shelter[title/abstract]

1. **Not genetics**

NOT (Genetic Phenomena[MeSH] OR DNA[title/abstract] OR RNA[title/abstract])

**Full search:**

((((((((adaptation[title/abstract] OR adapting[title/abstract] OR response[title/abstract] OR alert[title/abstract] OR implement*[title/abstract] OR awareness[title/abstract] OR strategy*[title/abstract] OR strategies[title/abstract] OR ”risk management”[title/abstract] OR ”risk management”[mesh] OR “emergency management”[title/abstract] OR “preparedness”[title/abstract] OR “disaster management”[title/abstract] OR warning*[title/abstract] OR “health promotion”[MeSH] OR “Health education”[MeSH] OR “communication campaign*”[Title/abstract] OR guideline*[Title/Abstract] OR recommendation*[Title/Abstract] OR “Health Services for the Aged”[mesh] OR “health facility planning”[MESH] OR “Public health surveillance”[MeSH] OR “home intervention”[title/abstract] OR “home care”[title/abstract] OR homecare[title/abstract] OR “home care services”[MESH] OR “patient care planning”[MESH] OR “Comprehensive Health Care”[mesh] OR outreach[title/abstract] OR “Patient care team”[MESH] OR multidisciplinary[title/abstract] OR “home visit”[title/abstract] OR “home assessment”[title/abstract] OR “patient management”[title/abstract] OR “social support”[MESH] OR shelter[title/abstract])) OR (cooling[title/abstract] OR “Air condition*”[title/abstract] OR “Air-condition*”[title/abstract] OR climatization[title/abstract] OR climatisation[title/abstract] OR ventilation[title/abstract] OR Fan[title/abstract] OR fans[title/abstract])) OR (media[title/abstract] OR communication*[title/abstract] OR audiovisual[title/abstract] OR helpline*[title/abstract] OR hotline*[title/abstract] OR telecommunication*[title/abstract] OR educat*[title/abstract] OR radio[title/abstract] OR television[title/abstract] OR TV[title/abstract] OR internet[title/abstract] OR campaign*[title/abstract] OR advert*[title/abstract] OR print*[title/abstract] OR “information campaign”[title/abstract] OR telemedicine[title/abstract] OR telehealth[title/abstract] OR telepharmac* OR e-health[title/abstract] OR ehealth[title/abstract] OR e-pharmac*[title/abstract] OR “social media”[mesh] OR “social media”[title/abstract] OR “social network*”[title/abstract] OR Twitter[title/abstract] OR Facebook[title/abstract] OR Linkedin[title/abstract] OR Pinterest[title/abstract] OR YouTube[title/abstract] OR "daily motion"[title/abstract] OR Yelp[title/abstract] OR Foursquare[title/abstract] OR "Google Circles"[title/abstract] OR Qype[title/abstract] OR Ello[title/abstract] OR Instagram[title/abstract] OR "Pip.io"[title/abstract] OR "Google Buzz"[title/abstract] OR Orkut[title/abstract] OR Tumblr[title/abstract] OR onesocialweb[title/abstract] OR asmallworld[title/abstract] OR bebo[title/abstract] OR myspace[title/abstract] OR folkdirect[title/abstract] OR virb[title/abstract] OR Friendster[title/abstract] OR storyofmylife[title/abstract])) OR (((“global warming”[Title/Abstract] OR “global warming”[MESH] OR climatic*[Title/Abstract] OR “climate change"[Title/Abstract] OR “climate change”[MESH] OR “Desert Climate”[mesh] OR “El Nino-Southern Oscillation”[Mesh] OR Microclimate[mesh] OR “Tropical Climate”[mesh]))))) NOT ((Genetic Phenomena[MeSH] OR DNA[title/abstract] OR RNA[title/abstract])))) AND ((((“ambient temperature”[title/abstract] OR “heat strain”[title/abstract] OR “heat exposure”[title/abstract] OR “heat stress”[title/abstract] OR Heating[mesh] OR “Heat Stress Disorders”[MESH] OR “hot temperature*”[title/abstract] OR “extreme heat”[MESH] OR “Heat stroke”[MESH] OR “heatstroke”[title/abstract] OR “heat index”[title/abstract] OR “heat episode”[title/abstract] OR “Heat Stress Disorders”[MESH] OR “heat event”[title/abstract] OR “Body temperature”[Mesh:noexp] OR “extreme temperature*”[title/abstract] OR “summer temperature*”[title/abstract] OR “Heat Exhaustion”[mesh] OR “summer weather”[title/abstract] OR “summer temperature*”[title/abstract] OR “heat wave”[title/abstract] OR “heatwave”[title/abstract] OR “indoor temperature”[title/abstract] OR “global temperature*”[title/abstract]))) NOT ((("Animals"[Mesh] NOT ("Animals"[Mesh] AND "Humans"[Mesh])))) NOT (“Chemicals and Drugs Category”[MeSH]) NOT ((("Plants"[Mesh] NOT ("Plants"[Mesh] AND "Humans"[Mesh])))))

## Web of Science search:

**Terms for heat and interventions:**

(TS=(ambient temperature OR heat strain OR heat exposure OR heat stress OR extreme heat OR Heat stroke OR heatstroke OR heat index OR heat episode OR heat event OR extreme temperature OR heat exhaustion OR heat wave OR heatwave OR hot temperature OR global temperature OR summer temperature OR summer weather OR outdoor temperature OR “Air conditioner*” OR “air conditioning”)) **OR**

(TI=(ambient temperature OR heat strain OR heat exposure OR heat stress OR extreme heat OR Heat stroke OR heatstroke OR heat index OR heat episode OR heat event OR extreme temperature OR heat exhaustion OR heat wave OR heatwave OR hot temperature OR global temperature OR summer temperature OR summer weather OR outdoor temperature OR “air conditioner*” OR “air conditioning”))

**AND LANGUAGE:** (English OR Chinese OR German) **AND DOCUMENT TYPES:** (Article)

**WC/SU**

(WC=(Medicine, Research & Experimental OR Public, Environmental & Occupational Health OR Health Care Sciences & Services OR Primary Health Care OR Film, Radio, Television OR urban studies OR Behavioral Sciences OR communication OR Infectious Diseases OR Planning & Development)) OR

(SU=(Communication OR Biomedical Social Sciences OR Health Policy & Services OR Public, Environmental & Occupational Health OR Urban Studies OR Research & Experimental Medicine OR Infectious Diseases OR Health Care Sciences & Services OR Behavioral OR Film, Radio & Television))

**NOT**

NOT TS=(wildlife OR animal OR fish OR flora OR conservation OR soil OR “heat shock protein” OR ”heat shock proteins” OR genetic OR “DNA” OR “RNA”)

**Indexes:**

Science Citation Index Expanded (SCI-EXPANDED) --1945-present

Social Sciences Citation Index (SSCI) --1945-present

Arts & Humanities Citation Index (A&HCI) --1975-present
