# Supplementary material for: Impacts of heat exposure in utero on long-term health and social outcomes: a systematic review
Source: BMC Pregnancy Childbirth. 2024 May 4;24:344. doi: 10.1186/s12884-024-06512-0 (PMC11069224; doi:10.1186/s12884-024-06512-0)
Supplement: Supplementary file 3 — Additional file 3. Author list for Climate and Heat-Health Study Group. Individual JBI risk of bias assessment forms, and excluded studies metadata from EPPI reviewer available on request. [file 12884_2024_6512_MOESM3_ESM.docx]

Supplementary Text 2

Climate Change and Heat-Health Study Group Author List:

Admire Chikandiwa (Department of Obstetrics and Gynaecology, School of Clinical Medicine, University of the Witwatersrand, Johannesburg, South Africa); Britt Nakstad (Institute of Clinical Medicine, Centre of Global Health, University of Oslo, Oslo, Norway and Department of Paediatrics and Adolescent Health, University of Botswana, Gaborone, Botswana); Caradee Y Wright (Department of Geography, Geoinformatics and Meteorology, Faculty of Natural and Agricultural Sciences, University of Pretoria, South Africa and Environment and Health Research Unit, South African Medical Research Council, Pretoria, South Africa); Lois Harden (Brain Function Research Group, Department of Physiology, University of the Witwatersrand, Johannesburg, South Africa); Nathalie Roos (Karolinska Institutet, Department of Medicine, Solna, Clinical Epidemiology Division, Stockholm, Sweden); Stanley M F Luchters (CeSHHAR Zimbabwe; International Centre for Reproductive Health and Department of Public Health and Primary Care, Ghent University, Ghent, Belgium); Cherie Part (Department of Public Health, Environments and Society, and the Centre on Climate Change and Planetary Health, London School of Hygiene and Tropical Medicine, London, UK); Minh Duc Pham(Burnet Institute, Melbourne, VIC, Australia, School of Public Health and Preventive Medicine and Department of Epidemiology and Preventive Medicine, Monash University, Melbourne, VIC, Australia); Ashtyn Areal(Leibniz Research Institute for Environmental Medicine, Düsseldorf, Germany); Marjan Mosalam Haghighi (Faculty of Medicine and Health, Central Clinical School, The University of Sydney, Sydney, New South Wales, Australia, The University of Sydney Charles Perkins Centre, Sydney, New South Wales, Australia and The Institute for Women, Children and their Families, Sydney Local Health District, Sydney, New South Wales, Australia); Albert Manyuchi (Global Change Institute, University of the Witwatersrand, Johannesburg, South Africa); Robyn Hetem(School of Animal, Plant and Environmental Sciences, Faculty of Science, University of the Witwatersrand, Johannesburg, South Africa); Melanie Boeckmann (Department of Global Health, Institute of Public Health and Nursing Research IPP, University of Bremen, Germany); Dilara Durusu( EIFFEL, Gelderland, Netherlands)
